# The Ophthalmoscope; Its Mode of Application Explained, Etc.

**Published:** 1859-01

**Authors:** 


ART. VI.—*The Ophthalmoscope; its Mode of Application Explained, and its Value shown in the Exploration of Internal Diseases affecting the Eye.* By JABEZ HOGG, Assistant Surgeon to the Royal Westminster Ophthalmic Hospital; Vice-President of the Medical Society of London, etc. etc. 8vo., pp. 107. London: Churchill, 1858.

THE brochure before us is a reprint, with various alterations and additions, of a paper read before the Medical Society of London, and published in the *London Lancet*, 1857, in Numbers XVI., XIX., XXI., of Vol. I., and VII. of Vol. II. Mr. Hogg has presented it to the public with the hope that it will serve to awaken the interest of the profession, and that it may serve as positive proof of the value of this instrument in diagnosis of diseases, which, before its appearance, were only known to us by subjective symptoms.

We have long been of the opinion that the ophthalmoscope, when used with judicious care and proper management, formed one of the greatest aids in the investigation of certain diseases of the internal eye, in regard to their prognosis and the treatment indicated in each particular case. In incipient or doubtful cases of cataract, amaurosis, and chronic inflammations of the deeper structures, and in foreign bodies in the vitreous humor or lens, its value is unquestioned; but it should, in our judgment, never be used in acute affections, in which the flood of light impinging on the parts would have the effect of producing a greater amount of irritation. For the proper use of this instrument, it is of course necessary that the observer should have as thorough a knowledge of the normal appearances of all the structures of the eye, as has the auscultator of the various sounds in the lungs in the healthy condition; without this we can expect to add nothing to our yet imperfect knowledge of diseased appearances. From the want of this precaution a certain amount of discredit has been brought upon the instrument by tyros and ignorant men; yet it has not, by any means, met with that amount of opposition as did the stethoscope when first introduced. The less complicated the construction of any instrument, the better it is adapted to our wants, and in none more than the ophthalmoscope should simplicity be the great object. The form preferred by the author is that of Mr. Anagnostakis, which consists of a concave mirror of ten inches focus, perforated in its centre. Directions are given for the employment of this, and the appearances of the healthy eye are pointed out.

An important point to be borne in mind by the observer is, as Mr. Hogg remarks, the influence produced on the retina by agents employed to dilate the pupil, thus rendering the diagnosis obscure or positively faulty. In regard to this he says:—

"I have repeatedly seen a drop of a weak solution of *atropine* produce in the healthy eye a very large amount of congestion of the retinoid vessels, more than sufficient to deceive the practiced eye of the surgeon, and which might well be mistaken for a diseased condition. On this account I have discontinued its use, and find no difficulty whatever, with the generality of patients, in causing sufficient dilatation of the pupil by simply placing them for a short time in a darkened room."

The "merit of the invention" Mr. Hogg gives to his countryman, Mr. Cumming, "who furnished the first idea for the ophthalmoscope, which is even now said to be the sole invention of the Germans, who were in truth the first to appreciate its worth, and publish it to the profession." "Twelve years ago he pointed out the existence of a reflection from the back of the human eye, and distinctly recognized the significance of the discovery." We must confess that we do not see the point of all this; why an idea should be confounded with an invention of undoubted German origin.

The section on the presence of cysterici in the eye, made up of Graeffe's cases, is interesting, and that on glaucoma is quite long and well written, incorporating within its limits several interesting cases. We must agree with the author in the fact that "the wood-cuts printed in the text imperfectly represent the appearances described;" he might have added that some are positively bad. The book, although it contains but few new facts, is an interesting one, and we would recommend it to the perusal of our readers.

As journalists, it is well known that we have always pursued a perfectly independent course in regard to criticisms on works sent us for review, commending wherever it may be considered just, and exposing faults without fear of consequences, as should be the duty of every conscientious reviewer. It is on this account, and not because of any personal ill-feeling to an author, that we never hesitate to comment prominently upon whatever may seem to deserve censure. We regret that before dismissing the present work, we have to point out a plagiarism on the part of a man who holds a respectable position in England. We had hoped that we had made an honest man of Mr. Hogg, but we find that he is inveterate in his literary thefts, a fact well known in Great Britain, and, in truth, told us by one of his own countrymen. Literary larceny is evidently deeply inlaid in his constitution, and we take it for granted that it would be as difficult to eradicate the evil from his system as it would be to eradicate the cancerous diathesis in a man whose organs are surcharged with scirrhous deposits.

To prove that our assertions are perfectly correct, we subjoin paragraphs from page 18 of Mr. Hogg's work and from page 509 of the first volume of this journal; the plagiarism is from a review of "Dixon on Diseases of the Eye," written by a gentleman of this city :—

Hogg's Ophthalmoscope :—

"Although I am willing to admit that the retina is not 'a merely passive object of examination' with this instrument, and that it is 'just the one tissue of the body which appreciates the intensity of the rays which fall upon it,' still I cannot agree with this gentleman in forbidding the use of the instrument for more than a few seconds at a time. The consequences dreaded from this concentration of light on the retina, have deterred many from using the instrument altogether; and this dread has even induced others to condemn it in toto, without ever giving it a trial.

I have employed it in cases of hyperæmia of the retina, retinitis, choroiditis, and posterior ophthalmitis, and have *never* seen any evil consequences follow its use; and in not more than half a dozen out of the whole number of cases in which I have used it have I found the patients complain of the intensity of the light, although the examinations were often continued long enough for me to sketch the diseased structures, and enable students to compare them after me."

REVIEW :—

"Although we are willing to admit that the retina is not 'a merely passive object of examination' with this instrument, and that it is 'just the one tissue of the body which appreciates the intensity of the rays which fall upon it,' still we cannot agree with the author in forbidding the use of the instrument for more than a few seconds at a time. The consequences dreaded from this concentration of light on the retina, have deterred many from using the instrument altogether; and this dread has even induced some to condemn it in toto, without ever giving it a trial.

We have employed it in cases of hyperæmia of the retina, acute retinitis, choroiditis, and posterior ophthalmitis, and we have *never* seen any evil consequence follow its use; and in not more than half a dozen out of the whole number of cases in which we have used it have we found the patients to complain of the intensity of the light, although the examinations were often continued long enough for us to sketch the diseased structures, or to enable a few professional friends to view them after us."

Is it not a little remarkable that Mr. Hogg should have appropriated the greater portion of this paragraph, without the slightest acknowledgment of any kind, from the very number of this journal in which he was so severely castigated a year and a half ago? To convince ourselves that our charge is not without foundation, we have consulted the numbers of the *Lancet* mentioned in the commencement of this notice, and do not find the paragraph in question. Mr. Hogg forgets the use of quotation marks, or apparently belongs to that happy class of individuals who flatter themselves that his countrymen never read American works, or who suppose that Americans themselves are ignorant of their own writings. We therefore abandon him to his fate as an irremediable and hopeless case.

